# Thyroid hormone insufficiency alters the expression of psychiatric disorder-related molecules in the hypothyroid mouse brain during the early postnatal period

**DOI:** 10.1038/s41598-021-86237-8

**Published:** 2021-03-24

**Authors:** Katsuya Uchida, Kentaro Hasuoka, Toshimitsu Fuse, Kenichi Kobayashi, Takahiro Moriya, Mao Suzuki, Norihiro Katayama, Keiichi Itoi

**Affiliations:** 1 Laboratory of Information Biology, Graduate School of Information Sciences, Tohoku University, Sendai, Japan; 2 Laboratory of Pharmacotherapy, Graduate School of Pharmaceutical Sciences, Tohoku University, Sendai, Japan; 3 National Institute of Occupational Safety and Health, Tokyo, Japan; 4 School of Pharmaceutical Sciences, Ohu University, Kōriyama, Japan; 5 Laboratory of Biomodeling, Graduate School of Information Sciences, Tohoku University, Sendai, Japan; 6 Department of Health and Nursing, Faculty of Health Sciences, Tohoku Fukushi University, Sendai, Japan

**Keywords:** Neuroscience, Endocrinology

## Abstract

The functional role of thyroid hormone (TH) in the cortex and hippocampus of mouse during neuronal development was investigated in this study. TH insufficiency showed a decrease in the expression of parvalbumin (PV) in the cortex and hippocampus of pups at postnatal day (PD) 14, while treatment with thyroxine from PD 0 to PD 14 ameliorated the PV loss. On the other hand, treatment with antithyroid agents in adulthood did not result in a decrease in the expression of PV in these areas. These results indicate the existence of a critical period of TH action during the early postnatal period. A decrease in MeCP2-positive neuronal nuclei was also observed in the cortical layers II–IV of the cerebral cortex. The brains were then stained with CUX1, a marker for cortical layers II–IV. In comparison with normal mice, CUX1 signals were decreased in the somatosensory cortex of the hypothyroid mice, and the total thickness of cortical layers II–IV of the mice was lower than that of normal mice. These results suggest that TH insufficiency during the perinatal period strongly and broadly affects neuronal development.

## Introduction

Thyroid hormone (TH) plays an important role in the development of the central nervous system (CNS), and TH insufficiency during the perinatal period negatively impacts the formation of dendritic trees in the cerebellum, production of myelin sheaths for myelinated nerves, and generation of new neurons in the hippocampus^1–4^. Recently, it was reported that TH insufficiency causes a decrease in the number of parvalbumin (PV)-expressing neurons, as a subpopulation of gamma-aminobutyric acid (GABA) interneurons, in the cortex and hippocampus^5–7^. Treatment with antithyroid agents from gestational day 6 to postnatal day (PD) 30 causes a dose-dependent decrease in PV-expressing neurons in the hypothyroid brain of rat; this phenomenon is ameliorated by the administration of thyroxine (T4) in these hypothyroid rats from PD 8 to PD 14^6^. Moreover, we have reported that the number of PV-expressing neurons is low in the cerebral cortex of growth-retarded mice used as a mouse model of hypothyroidism. This phenomenon was ameliorated by treatment with tri-iodothyronine (T3) from PD 0 to PD 20^8^. These reports suggest the existence of a critical period during the early postnatal period wherein TH is sensitive to PV expression. The hypothyroid state and a mutation of the TH receptor alpha gene leads to defects in GABA-mediated cortical network activity, such as a reduction in inhibitory function and gamma oscillation frequency^7^. This finding implies that mental retardation in cretinism is partly caused by dysfunction of the cortical network activity. On the other hand, a decrease in the number of PV-expressing neurons is commonly observed not only in hypothyroid animal models but also in the postmortem brain of patients with psychiatric disorders, such as schizophrenia and autism^9,10^. Rett syndrome (RTT), which results from a mutation of the methyl-CpG-binding protein 2 (MeCP2), is considered to be an autism spectrum disorder^11,12^, and the expression of PV is significantly decreased in the cortical layers II–V of MeCP2 deficient mice^13^. Interestingly, a cohort study showed that patients with RTT exhibit thyroid function abnormalities, with 17.7% of the patients showing an increase in free-thyroxine, whereas 10.2% of the patients exhibited subclinical hypothyroidism^14^. Although these patients with RTT who manifested thyroid function abnormalities had MeCP2 mutations, no study has reported a histological analysis of MeCP2 expression in the hypothyroid brain. Accordingly, we herein report that the expression of psychiatric disorders-related molecules in the hypothyroid brain of mice is altered by TH insufficiency during the early postnatal period.

## Materials and methods

### Animals

Mice (C57 BL/6J) were maintained under conditions of controlled temperature (23 ± 1 °C), relative humidity (50 ± 1%), and lighting (07:00–19:00 h). The control mice were fed laboratory chow (Labo MR Breeder; Nosan, Yokohama, Japan) with ad libitum tap water. To generate hypothyroid mice model, pregnant mice were administered antithyroid agents (1% potassium perchlorate and 0.05% methimazole in tap water) from gestational day 17 to postnatal day 14. From postnatal day 15 onward, the dams and pups were given normal water. To observe the effects of exogenous thyroid hormone on the expression of PV mRNA, the mice were injected with thyroxine (T_4_; 20 ng/g body weight; Sigma-Aldrich Co., MO, USA) from PD 0 to PD 14.

All animal experiments were conducted in accordance with international standards on animal welfare according to the National Institutes of Health Guide for the Care and Use of Laboratory Animals, the ARRIVE guidelines, and the Animal Experiment Guidelines of the Institutes for Animal Experimentation at Tohoku University. The protocol for animal experiments was approved by the Center for Laboratory Animal Research, Tohoku University (reference number: 2016IsA-004-1).

### Hormone assay

Serum-free thyroxine levels were assayed from the trunk or circulating blood, which were collected at PD 14, 15, 21, 30, and 90. After collection, the specimens were stored at room temperature for 30 min, and thereafter at 4 °C overnight. The blood specimens were then centrifuged at 800×*g* for 15 min at 4 °C, and serum was collected in a tube. Serum samples were then assayed using the Human Total T4 ELISA Kit (Alpha Diagnostic International Inc. TX, USA).

### Tissue preparation and Immunohistochemistry

Under isoflurane anesthesia, mice were deeply anesthetized with three anesthetic agents: 3 µg medetomidine, 40 µg midazolam, and 50 µg butorphanol per 10 g body weight. The mice were transcardially perfused with 0.9% saline, and then by 4% paraformaldehyde (PFA) in 0.1 M phosphate buffer (PB; pH 7.4). Then, the brains were surgically obtained and fixed in 4% PFA at 4 °C for 6 h and immersed in 30% sucrose in 10 mM PB (pH 7.4) at 4 °C for 48 h until the brains sank to the bottom of the tube. Thirty micrometer-thick sections were serially cut on a cryostat, and the free-floating sections were rinsed in 10 mM phosphate-buffered saline (PBS; pH 7.4). For MeCP2 immunostaining, after washing with PBS containing 0.1% (w/v) Triton X-100 (PBS-T), brain sections were subjected to antigen retrieval with citrate buffer (pH 6.0). After washing with PBS-T, the sections were incubated for 30 min with 1% normal goat serum diluted in PBS-T. The sections were then incubated overnight at 4 °C with rabbit anti-MeCP2 (clone No. D4F3XP, 1:500, catalogue No. 3456, Cell Signaling Technology, MA, USA). Thereafter, the sections were treated with 1% H_2_O_2_ to inactivate endogenous peroxidases. After another wash with PBS-T, the sections were incubated with biotinylated goat anti-rabbit IgG (1:1000; catalogue No. BA-1000-1.5, Vector Laboratories Inc., CA, USA) for 2 h at room temperature, rinsed with PBS-T, and incubated with ABC solution (VECTASTAIN elite ABC standard kit, catalogue No. PK-6100, Vector Laboratories Inc.) for 1 h at room temperature. Finally, the sections were visualized with 3,3′-diaminobenzidine (DAB). For CUX1 staining, after washing with PBS-T, sections were incubated for 30 min with 1% normal donkey serum diluted in PBS-T. The sections were then incubated overnight at 4 °C with rabbit anti-CUX1 (CDP, M-222, 1:500; catalogue No. sc-13024, Santa Cruz Biotechnology Inc., CA, USA), a marker for cortical layers II–IV^15^. After washing with PBS-T, the sections were incubated with biotinylated goat anti-rabbit IgG (1:1000; Vector Laboratories Inc.) or Alexa-conjugated goat anti-rabbit IgG (Alexa Fluor 594) (1:1000; catalogue No. ab150080, Abcam plc, cambridge, UK) for 2 h at room temperature. After incubation with biotinylated goat anti-rabbit IgG, the sections were incubated with ABC solution and then visualized with DAB.

### Pulse-chase analysis

To confirm whether PV-expressing neurons are spontaneously generated in the cortex and hippocampus during the early postnatal period, we performed pulse-chase analysis using 5-bromo-2′-deoxyuridine (BrdU). At PD 0, 7, and 14, mice were administered a single dose of BrdU (50 mg/kg of body weight, intraperitoneal; Sigma-Aldrich Co.). One month after BrdU injection, the mice were euthanized and their brains were collected as described above. Brain sections were made as aforementioned and antigen retrieval was performed using citrate buffer (pH 6.0). After washing with PBS-T, the sections were incubated for 30 min with 1% normal donkey serum diluted in PBS-T. The sections were then incubated overnight at 4 °C with mouse anti-PV (clone No. PARV-19, 1:1000, catalogue No. P3088, Sigma-Aldrich Co., Supplemental Fig. 1) and rat anti-BrdU (clone No. BU1/75(ICR1), 1:250, catalogue No. ab6326, Abcam plc). After washing with PBS-T, the sections were incubated with Alexa 555-conjugated donkey anti-mouse IgG (1:1000; catalogue No. A-31570, Thermo Fisher Scientific, MA, USA) and Alexa 488-conjugated donkey anti-rat IgG (1:1000; catalogue No. A-21208, Thermo Fisher Scientific) for 2 h at room temperature.

### In situ hybridization

The sequence of the PV probe (Probe RPS_060524_04_D07) was selected according to the Allen Mouse Brain Atlas. This sequence was amplified by polymerase chain reaction (PCR) and cloned with a TA PCR cloning kit (pTAC-2, BioDynamics Laboratory Inc., Tokyo, Japan). PV anti-sense probe was labeled with digoxigenin (DIG), and the anti-sense probe was hybridized to the brain sections using the general method of in situ hybridization. The probes were then detected with alkaline phosphatase-conjugated anti-DIG Fab fragments (1: 5000, catalogue No. 11093274910, Roche, Sigma-Aldrich Co.) and visualized with NBT/BCIP (Roche, Sigma-Aldrich Co.).

### Thionin stain

To investigate the developmental differences in cortical layers between normal and hypothyroid mice, brain sections were stained with thionin, which detects Nissl bodies (i.e., rough-surface endoplasmic reticulum and polysome). The total area of cortical layers II–IV, which included the somatosensory, auditory, and visual cortices, was measured using ImageJ (NIH, USA). The region of interest was divided into 3 areas (ROI 1: − 0.70 to − 1.34 mm, ROI 2: − 1.34 to 1.82 mm, and ROI 3: − 1.82 to − 2.30 mm from the bregma) along the rostrocaudal axis, and the areas were compared between normal and hypothyroid animals.

### Relative quantitative PCR (qPCR)

Total RNA was extracted from tissue homogenates using ISOGEN II (Nippon Gene, Toyama, Japan), and cDNA was synthesized using PrimeScript II 1st strand cDNA Synthesis Kit (TAKARA, Shiga, Japan). Each cDNA strand was amplified with Taq polymerase for qPCR (THUNDERBIRD qPCR Mix, TOYOBO CO., LTD, Osaka, Japan), and the fluorescence intensity of each sample was detected using a LightCycler (Roche, Basel, Switzerland). Relative quantitative in real-time RT-PCR was performed using Pfaffl’s method^16^. Each primer sequence is displayed in Table 1.

**Table 1:** Primer set for qPCR.

GAPDH (forward)	5′-GGCATTGCTCTCAATGACCA-3′
GAPDH (reverse)	5′-TGTGAGGGAGATGCTCAGTG-3′
PV (forward)	5′-TTCTGAAGGGCTTCTCCTCA-3′
PV (reverse)	5′-TTCTTCAACCCCAATCTTGC-3′
MeCP2 (forward)	5′-CATACATAGGTCCCCGGTCA-3′
MeCP2 (reverse)	5′-CAGGCAAAGCAGAAACATCA-3′

### Statistical analysis

For statistical analysis, we used GraphPad Prism 7 software (GraphPad Software, Inc.). The Student’s unpaired *t* test was performed to compare the body weight between experimental groups. One-way analysis of variance (ANOVA) was performed to investigate the effect of antithyroid agents and exogenous T4 on the expression of PV mRNA in the mouse brain. If there was a significant difference between groups, Tukey’s multiple comparison test was carried out as a post-hoc analysis. Two-way ANOVA was used to investigate the change in serum T_4_ levels and in the area of cortical layers after treatment with antithyroid agents. If there was a significant interaction between the factors, Sidak’s multiple comparisons test was carried out as a post-hoc analysis. All data are shown as mean ± standard error of mean. Statistical differences were considered significant at p < 0.05.

## Results

**Figure 1:**
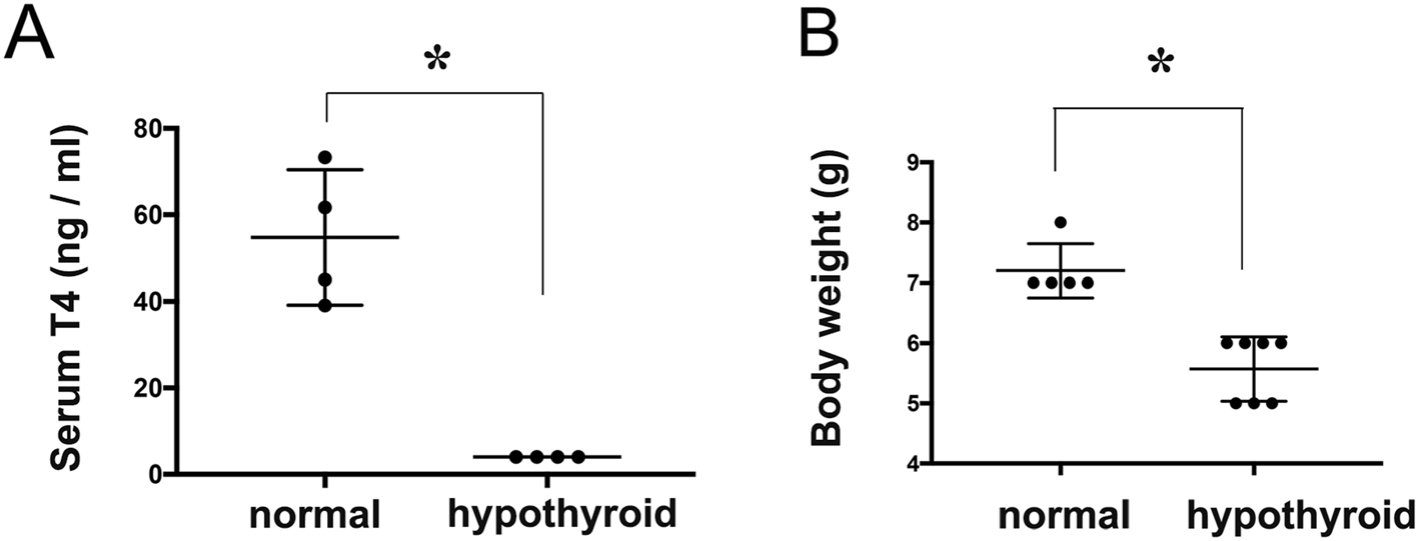
Effects of antithyroid agents on serum thyroxine levels and body weight of juvenile mice. (**A**) Graph shows the levels of serum thyroxine (T_4_) in mice at postnatal day 14. Each group, n = 4; *p < 0.0001. (**B**) Graph shows the body weight of mice at postnatal day 14. Hypothyroid group, n = 7; control group, n = 5. *p < 0.002.

**Figure 2:**
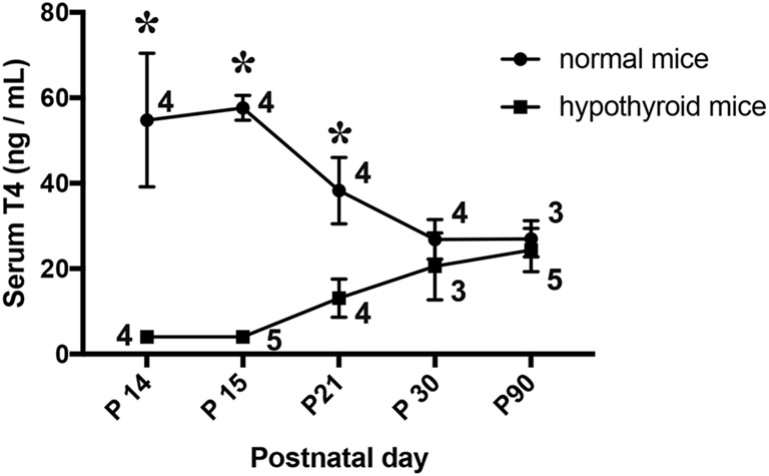
Change in serum T_4_ after termination of treatment with antithyroid agents. Replacement of drinking water that included antithyroid agents with normal water at PD 14. Serum T_4_ levels in hypothyroid mice caught up to the same level as normal mice 2 weeks later. Arabic numerals in line graphs indicate the number of animals. *p < 0.0001 vs. normal mice.

Antithyroid agents resulted in a dramatic decrease in the level of serum thyroxine (normal 54.79 ± 7.8 ng/ml, n = 4 vs. hypothyroid not detected. < 4 ± 0 ng/ml, n = 4, p < 0.0001) of juvenile mice at PD 14, in addition to low weight (normal 7.2 ± 0.2 g, n = 5 vs. hypothyroid 5.5 ± 0.2 g, n = 7, p < 0.002) (Fig. 1). After replacement of drinking water that included antithyroid agents with normal water, the levels of serum thyroxine gradually increased and reached the same level as that in normal mice approximately 16 days later (PD 30) (Fig. 2). Results of two-way ANOVA indicated a significant interaction between intergroup (normal vs. hypothyroid) and intragroup variability (time course) (F (4, 30) = 25.56, p < 0.0001), as well as the main effects of intergroup (F (1, 30) = 169.4, p < 0.0001), but not of time course (F (4, 30) = 1.553, p = 0.2123). Sidak’s multiple comparisons test indicated that the levels of serum T4 were significantly lower in the hypothyroid groups (PD 14 to 21) than in the normal groups (p < 0.0001), and that the levels of T4 in the hypothyroid groups recovered at P 30 (approximately 2 weeks after termination of the treatment with antithyroid agents).

**Figure 3:**
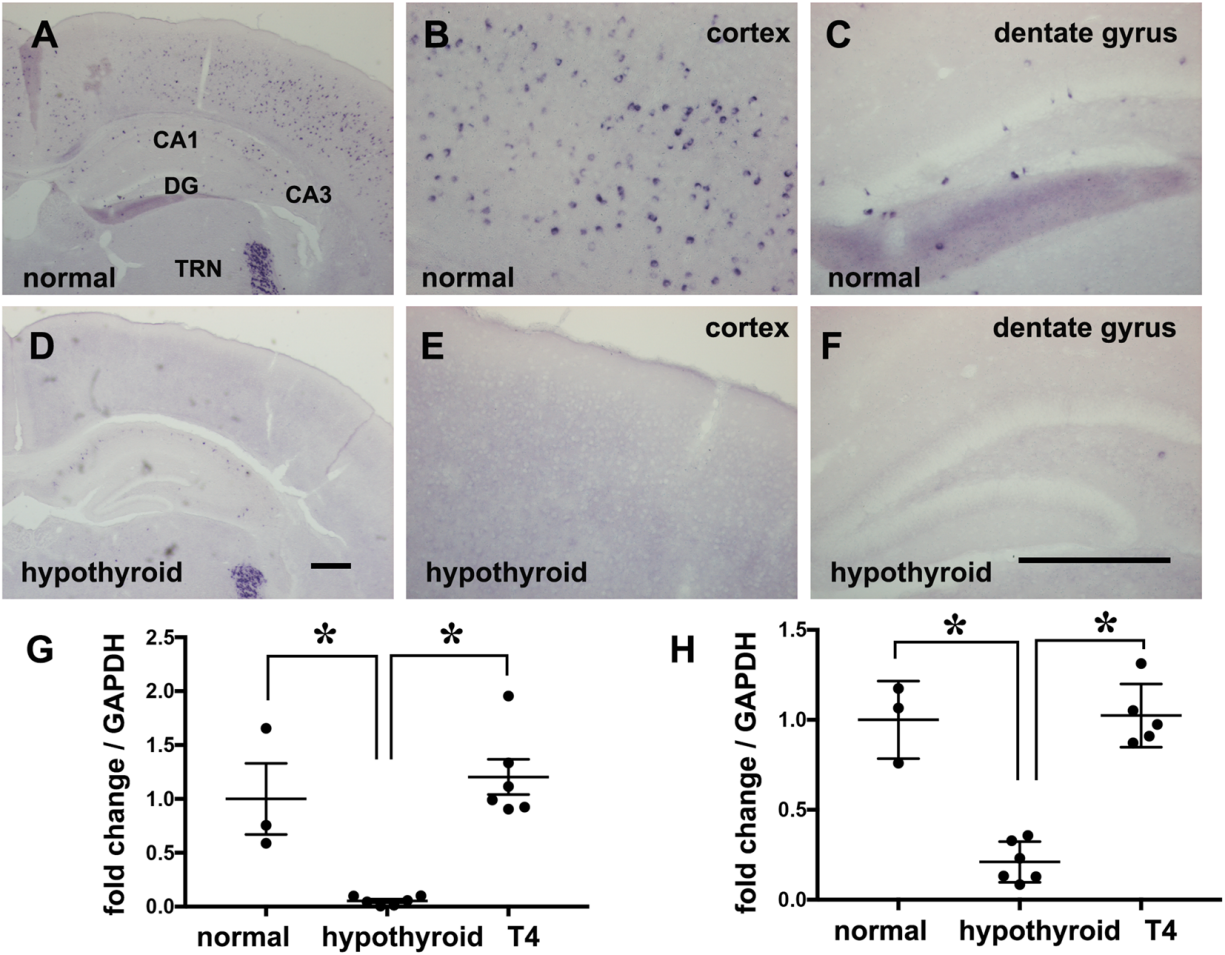
Expression of PV mRNA in the cerebral cortex and hippocampus at PD 14 after termination of treatment with antithyroid agents. (**A**–**C**) Expression of PV mRNA in the cerebral cortex and hippocampus of normal mice. (**B**,**C**) Magnified images of the cerebral cortex (**B**) and the dentate gyrus of the hippocampus (**C**) from (**A**). (**D**–**F**) Expression of PV mRNA in the cerebral cortex and hippocampus of hypothyroid mice. (**E**,**F**) Magnified images of cerebral cortex (**E**) and the dentate gyrus of the hippocampus (**F**) from (**D**). Scale bar = 400 µm. *CA* Cornu Ammonis, *DG* dentate gyrus, *TRN* thalamic reticular nucleus. (**G**,**H**) Graphs show expression of PV mRNA in the cortex (**G**) and hippocampus (**H**) using qPCR. *p < 0.01: normal vs. hypothyroid (cortex), p < 0.001: hypothyroid vs. T4 (cortex). *p < 0.001: normal vs. hypothyroid (hippocampus), p < 0.001: hypothyroid vs. T4 (hippocampus).

After termination of antithyroid agents, in situ hybridization was performed to confirm the expression of PV mRNA in the cortex and hippocampus between the experimental groups. Although PV mRNA was broadly distributed in the cortical layers II–VI of the cerebral cortex of normal mice (Fig. 3A,B), TH insufficiency during E 17.5 to PD 14 resulted in the loss of PV expression in these areas (Fig. 3D,E). In the hippocampus, PV signals were observed in the hilus, granular cell layer, and cornu ammonis (Fig. 3A,C). These signals decreased dramatically following treatment with antithyroid agents (Fig. 3D,F); as similar observation was noted in the cortex. In both the cortex and hippocampus, qPCR analysis indicated that the levels of PV mRNA expression were decreased by treatment with an antithyroid agent during the perinatal period, while the expression levels recovered upon exogenous T4 treatment from just after birth to PD 14 (Fig. 3G,H). One-way ANOVA yielded significant differences between groups in both the cortex and hippocampus. A post-hoc test (Tukey’s multiple comparison test) indicated that expression levels of PV mRNA were significantly lower in hypothyroid mice than in normal mice (cortex: p < 0.01, hippocampus: p < 0.001) and that treatment with T4 during PD 0 to 14 markedly inhibited the decrease in the expression of PV mRNA in the hypothyroid mice (cortex: p < 0.001, hippocampus: p < 0.001). On the other hand, treatment with antithyroid agents had no effect on PV expression in the dam (data not shown).

**Figure 4:**
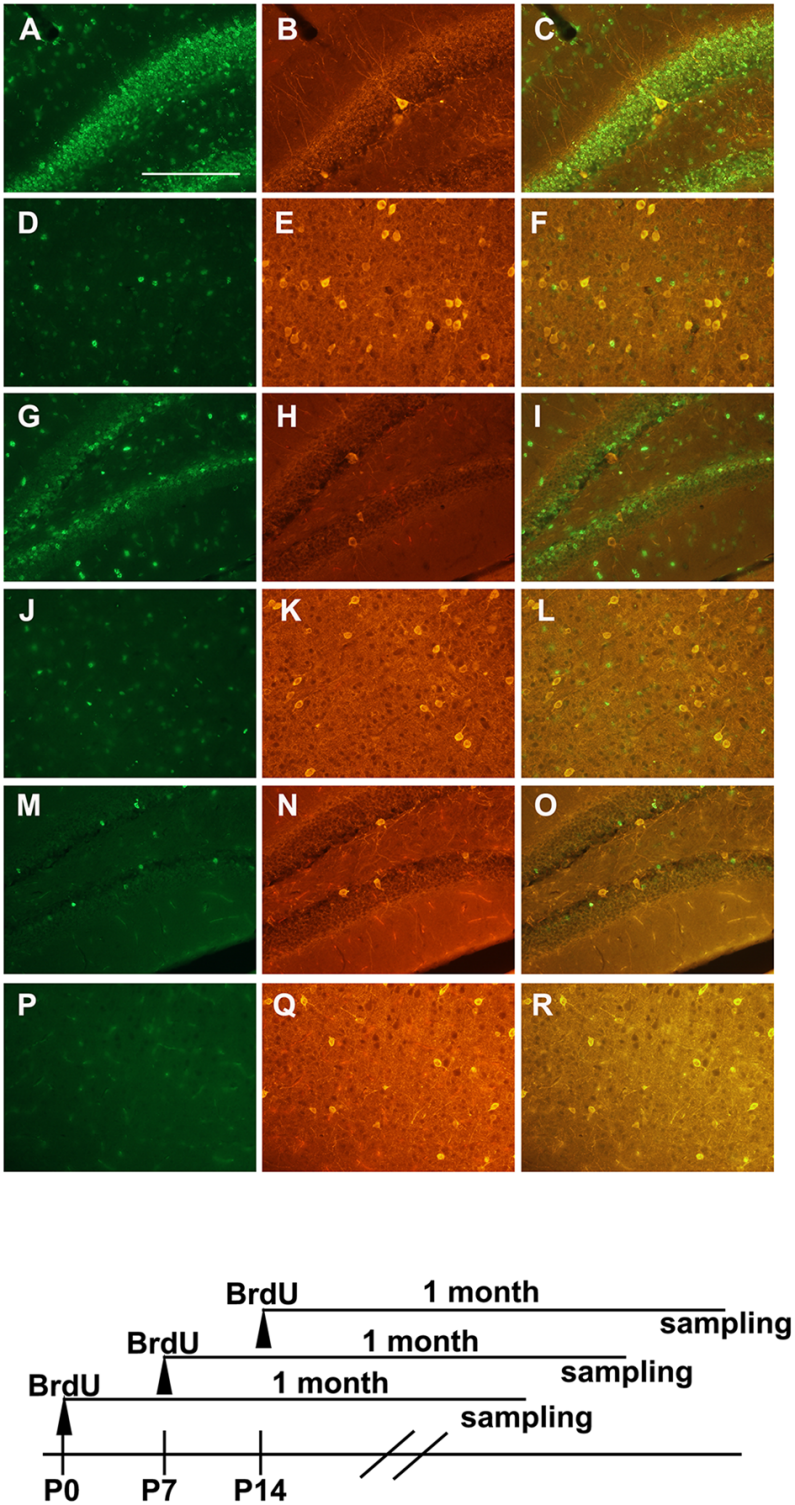
Validation of neurogenesis of PV neurons by BrdU pulse-chase experiment. Photomicrographs show immunostaining of BrdU (green), PV (orange), and merged image (most right panels) one month after BrdU injection. (**A**–**F**) Immunostaining images of the dentate gyrus of the hippocampus (**A**–**C**) and cerebral cortex (**D**–**F**) of mice injected with BrdU on PD 0. (**G**–**L**) Immunostaining images for the dentate gyrus of the hippocampus (**G**–**I**) and cerebral cortex (**J**–**L**) of mice injected with BrdU on PD 7. (**M**–**R**) Immunostaining images for the dentate gyrus of the hippocampus (**M**–**O**) and cerebral cortex (**P**–**R**) of mice injected with BrdU on PD 14. Scale bar = 200 µm.

To confirm the day of PV neuron formation, pulse-chase analysis using BrdU was performed. At PD 0, PD 7, and PD 14, mice were injected with BrdU, and BrdU-incorporated cells were observed in the cortex and hippocampus one month after BrdU injection (Fig. 4). As is well known, many proliferating cells that incorporated BrdU were observed at earlier stages of brain development. Thus, BrdU-incorporated cells were mostly observed in the hippocampus at PD 0, and the number of BrdU-labelled cells gradually decreased with age (Fig. 4A,G,M). The number of BrdU-labelled cells was lower in the cerebral cortex than in the hippocampus, and these cells were rarely detected in the cortex on PD 14. Regarding colocalization of BrdU and PV, PV-neurons did not have BrdU in their nuclei in any of the groups (Fig. 4).

**Figure 5:**
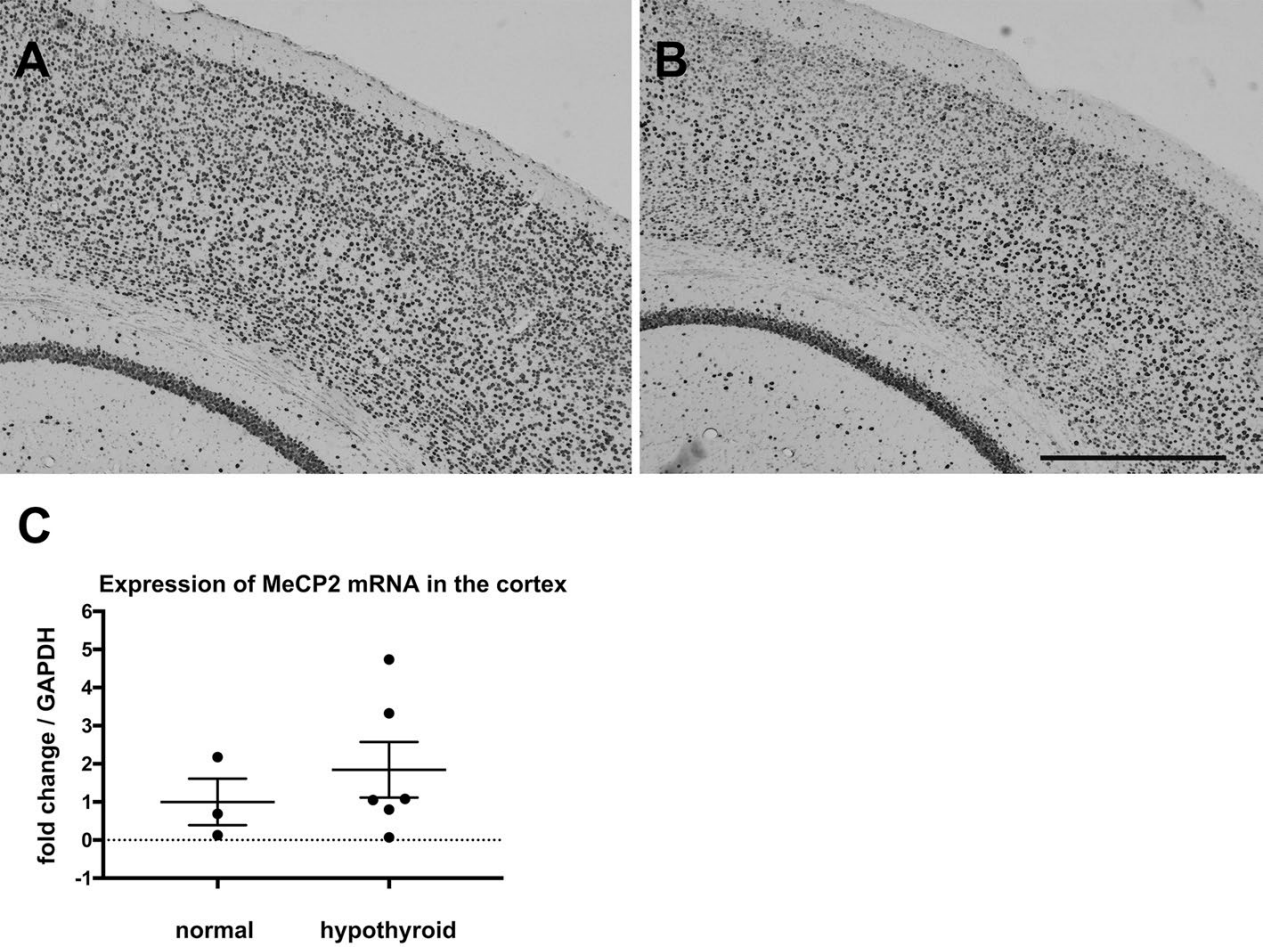
Expression of MeCP2 in the cerebral cortex of mice. (**A**,**B**) Photomicrographs show immunostaining of MeCP2 in the cerebral cortex of normal (**A**) and hypothyroid mice (**B**) at PD 14, respectively. Scale bar = 500 µm. (**C**) Graph shows the results of qPCR for MeCP2 mRNA in the cerebral cortex of mice at PD 14.

MeCP2 signals were ubiquitously observed in the cortex and hippocampus of the mouse brain, and the immunostaining was confined to the cell nucleus (Fig. 5A). TH insufficiency during the perinatal period resulted in a decrease in signal intensity of MeCP2 in the cerebral cortex, especially in layers II–IV and layer V of cortex at PD 14 (Fig. 5B). Heterogeneity of MeCP2 staining was also observed in some areas of the cerebral cortex in the hypothyroid group (Supplemental Fig. 2). In the hippocampus, there were no significant differences between the normal and hypothyroid groups (data not shown). On the other hand, qPCR indicated no significant differences in the expression of MeCP2 mRNA in the cortex between any of the groups (Fig. 5C). At age 3 months, the signal intensity of MeCP2 was equivalent between the normal and hypothyroid groups, and mRNA expression levels were also not significantly different between the groups (data not shown).

**Figure 6:**
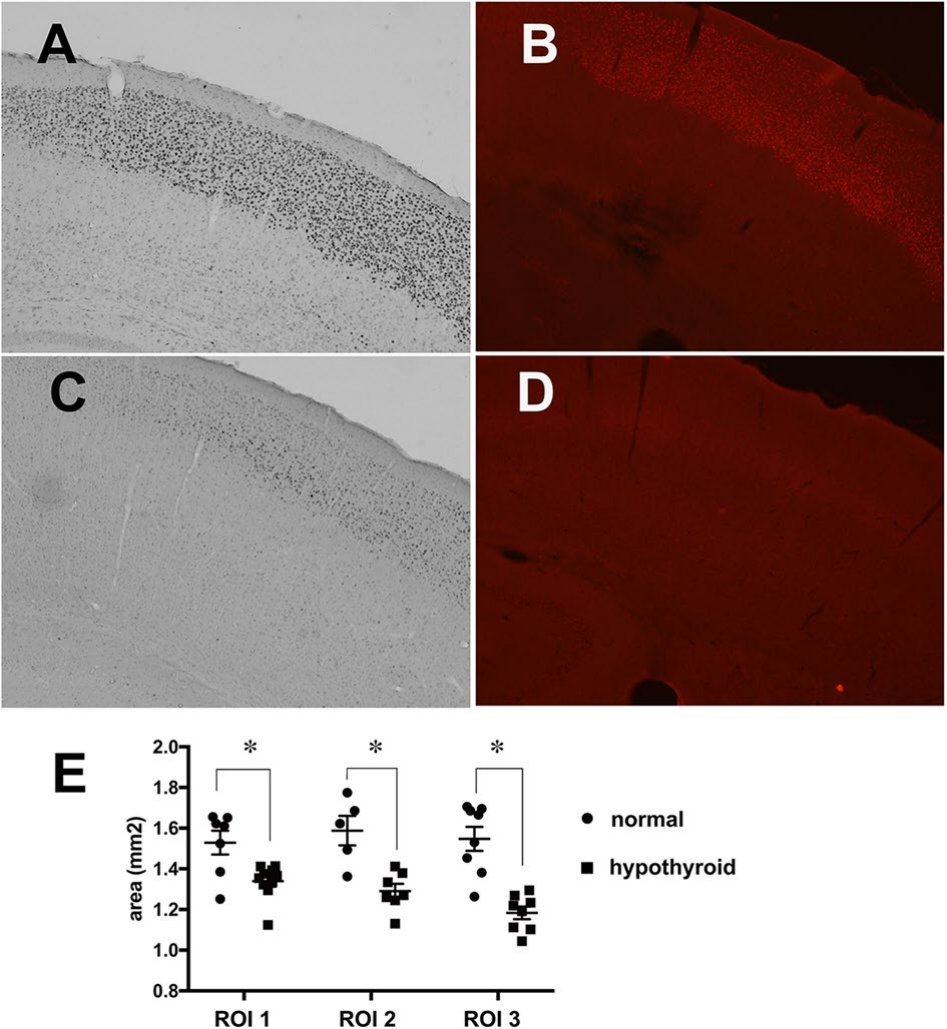
Expression of CUX1 in layers II–IV of the cerebral cortex in mice. (**A**–**D**) Photomicrographs show immunostaining of CUX1 in the cerebral cortex of normal (**A**,**B**) and hypothyroid mice (**C**,**D**), respectively. (**A**) and (**C**) at PD 14; (**B**) and (**D**) at 3-months of age. Scale bar = 500 µm. (**E**) Graph shows the areas of cortical layers II–IV in normal (circle) and hypothyroid mice (square) at 3-months of age. ROI 1: − 0.70 mm to − 1.34 mm, ROI 2: − 1.34 mm to 1.82 mm, ROI 3: − 1.82 mm to − 2.30 mm from the bregma.

Furthermore, CUX1 staining revealed that the total thickness of cortical layers II–IV of hypothyroid mice was lower than that of normal mice at PD 14 (Fig. 6A,C). At age 3 months, the expression levels of CUX1 were still lower in the hypothyroid group than in the normal group, although the expression of PV and MeCP2 was equivalent between the normal and hypothyroid groups (Fig. 6B,D). Thionin staining revealed that layers II–IV were thinner in hypothyroid mice than in normal mice across a range of areas, including the somatosensory, auditory, and visual cortices (Fig. 6E).

## Discussion

We show for the first time that TH insufficiency during the perinatal period induces changes in the expression of psychiatric disorder-related molecules (MeCP2, CUX1), in addition to the already-known molecule, PV.

Hypothyroidism causes a decrease in body weight^17,18^. In the present study, treatment with antithyroid agents also led to growth retardation in mice. Since congenital hypothyroidism not only leads to growth retardation but also results in structural disorder of the brain^1,2,19^, TH during the perinatal period is important for normal development of the organism. In our experiments, mice were treated with antithyroid agents from gestational day 17. We cannot describe definitely when the antithyroid agents start to have significant effect on pups. However, Hassan et al., indicated that the effects of MMI on thyroid hormones is observed from day 4 after treatment with MMI^20^. Further, MMI is excreted in a relatively higher concentration in human milk^21^. According to these reports, pups may be affected by antithyroid drugs form gestational day 21/postnatal day 1. Serum TH levels during the neonatal period transiently increase at the end of the second postnatal week^19,22,23^. This suggests the possibility that normal neural development may be controlled by a TH surge after birth. Indeed, Fishman et al. have shown that the elimination of the peak in TH levels by treatment with pentobarbital induces neural damage in the cerebellum^19^. Since pentobarbital can interfere with the thyroid system^24^, the result indicates that TH surge at the end of the second postnatal week plays an important role in normal neuronal development. On the other hand, Gilbert et al. have shown that treatment with exogenous T_4_ in antithyroid agent-induced hypothyroid rats during PD 8 to 14 prevented the decrease in the number of PV-expressing neurons^6^. In our previous report, whereas continuous treatment of hypothyroid mice during PD 0 to 14 with T_3_ inhibited the decrease in the number of PV neurons, treatment beyond PD 14 had no effect on resurgence of the number of PV-expressing neurons in the mouse brain^8^. Thus, these results suggest that the developing brain has a critical period of sensitivity to TH, and that the critical period exists within the first 14 days of life. However, presently, we cannot conclude whether normal neural development is achieved by the transient peak in TH levels or by the abundance of TH. The phenomenon that causes a decrease in the expression of PV is commonly observed in rodents^5,8,25^. The symptoms have not only been observed in an antithyroid agent-induced hypothyroid model but also in genetically engineered mice presenting with TH-related impairments, such as dysfunctions of the TH receptor and TH transporter^7,26,27^. These findings thus indicate that TH regulates the expression of PV.

PV-expressing interneurons are generated from the medial ganglionic eminence, and these immature neurons migrate tangentially to their respective destination areas^28–30^. In the present study, we confirmed the role of TH on the cell lineage of PV neurons using pulse-chase analysis with BrdU. From birth to PD 14, no co-localization of BrdU and PV was observed in the cortex and hippocampus. These results suggest that the mitotic phase of PV progenitor cells is already terminated, which is in agreement with previous reports^30,31^. In the cortex and hippocampus, developmental expression of PV mRNA is observed, starting approximately from PD 10, and the expression level gradually increases into adulthood^32^. Thus, TH may promote PV gene expression. On the other hand, expression of GAD67 (GAD1) mRNA remained intact in hypothyroid mice (Supplemental Fig. 3). In addition, treatment of rats with antithyroid agents had no effect on GAD67 immunostaining in the cortex and hippocampus^6^. Therefore, a significant reduction in PV-expressing neurons in the hypothyroid brain may reflect an impairment of transcription of the PV gene in GABAergic interneurons, without decreasing the total number of GABA neurons. Further studies are needed to elucidate the molecular mechanisms underlying the direct or indirect action of TH on the transcription of PV genes.

Immunoreactivity of MeCP2 was significantly decreased in cortical layers II–IV of hypothyroid mice. Bunker et al. have also reported that neonatal exposure to PTU in male rats has a significant effect on the expression of MeCP2 in the adult liver^33^. In our study, immunoreactivity of MeCP2-expressing nuclei in the cortical layers II–IV of hypothyroid mice was markedly lower than that in control mice, whereas MeCP2 mRNA expression showed no significant difference. In the liver, MeCP2 transcripts are significantly low, while its translated products are at a higher levels than that in controls^34^. Although we cannot explain why the transport of transcripts and translated products differs between organs, these results suggest that TH has a significant effect on the expression of MeCP2. On the other hand, MeCP2 knockout model shows a decrease in TH-related gene expression^35^, and some patients with MeCP2 mutations show a decrease in serum T4 or an increase in thyroid-stimulating hormone (subclinical hypothyroidism)^14,36^. These reports suggest that TH-related gene expression is altered in patients with RTT and TH can play an important role in RTT clinical phenotype. Briefly the reports show MeCP2 gene regulates TH-related gene. The further studies are needed whether TH directly or indirectly regulates MeCP2 expression.

Retardation of PV expression in the cortical layers of MeCP2-deficient mice is similar to that of our hypothyroid mice model^13^. The two molecules may be in a complementary relationship to express normal functions in the cytoplasm of inhibitory neurons. In fact, failure of MeCP2 in GABAergic interneurons targeting the vascular inhibitory amino acid transporter was shown to promote RTT-like behavior^37^, and PV neuron-restricted MeCP2 dysfunction indicated motor, sensory memory, and social deficits^38^. Thus, these phenotypes may result from the activity of MeCP2 and PV. We believe that the MeCP2 molecule, in addition to PV, participates in behavioral dysfunction with hypothyroidism.

The observation of a decrease in CUX1 in cortical layers II–IV compelled us to analyze a comparison of somatosensory areas between experimental groups. The total thickness of cortical layers II–IV of hypothyroid mice was lower than that of normal mice. Taking into account our findings with previous studies, thyroid hormones may play an important role in normal corticogenesis. Consistent with this notion, in rat studies, maternal hypothyroidism was shown to bring about impairment of corticogenesis as a result of reducing the rate of neuronal proliferation (neurogenesis) and/or failure of radial migration^39–41^. Further, Calikoglu et al. reported that the development of primary somatosensory cortex in layer IV of mice was delayed by propylthiouracil treatment during the perinatal period^23^. This phenomenon is ameliorated by treatment of exogenous T4 to pregnant dams^39,40^, and treatment with antithyroid agents for adult mice did not affect the volume density of the cortex^23^. Thus, cortical neurons may strongly require the presence of TH during the developmental stage. In fact, patients who experienced insufficient maternal TH in utero indicate a morphogenetic abnormality in the human cortex^42^, and this finding using magnetic resonance imaging in the human brain warrants the results of rodent studies.

A continuous decrease in the transcription factor CUX1 was observed in cortical layers II–IV of hypothyroid mice. CUX1 regulates dendritic branching and spine morphology in the cortex, and inhibition of this transcription factor by RNA interference causes dysgenesis of dendritic spines and branching^43,44^. On the other hand, hypothyroidism also indicates dysgenesis of dendritic spines and branching^45–48^. Therefore, the morphogenetic abnormalities observed in hypothyroidism may result from hypoactivity of CUX1. As an example of the functional association of CUX1 with the thyroid hormone, a decrease in the expression of the reelin protein in the cortical plate is observed in CUX1 knockout mice and in hypothyroid animals^49,50^. Reelin is a secreted glycoprotein that regulates the radial migration of cortical neurons and branching^51–53^. Thus, we consider that morphological impairment of microstructures, which is induced in CUX knockout and hypothyroid mice, is engendered by dysfunction of common molecular mechanisms.

Our findings revealed that thyroid hormone insufficiency during the perinatal period resulted in a decrease in the expression levels of MeCP2 and CUX1, in addition to that of the already-known PV. Given that decreased levels of these molecules have been observed in postmortem autistic brains and in genome-wide studies^54–58^, clues for the leading cause of autism might be provided by research on the central nervous system in hypothyroidism.

## Supplementary Information

Supplementary Information.
