# Supplementary material for: Thyroid hormone insufficiency alters the expression of psychiatric disorder-related molecules in the hypothyroid mouse brain during the early postnatal period
Source: Sci Rep. 2021 Mar 24;11:6723. doi: 10.1038/s41598-021-86237-8 (PMC7990947; doi:10.1038/s41598-021-86237-8)
Supplement: Supplementary file 1 — Supplementary Information. [file 41598_2021_86237_MOESM1_ESM.pdf]

Supplementary material and data

Thyroid hormone insufficiency alters the expression of psychiatric disorder-related molecules in the hypothyroid mouse brain during the early postnatal period

Katsuya Uchida [1*], Kentaro Hasuoka [2], Toshimitsu Fuse [1], Kenichi Kobayashi [3], Takahiro Moriya[4], Mao Suzuki [5], Norihiro Katayama [5], Keiichi Itoi [6]

[1] Laboratory of Information Biology, Graduate School of Information Sciences, Tohoku University, [2] Laboratory of Pharmacotherapy, Graduate School of Pharmaceutical Sciences, Tohoku University, [3] National Institute of Occupational Safety and Health, Japan, [4] School of Pharmaceutical Sciences, Ohu University, [5] Laboratory of Biomodeling, Graduate School of Information Sciences, Tohoku University, [6] Department of Health and Nursing, Faculty of Health Sciences, Tohoku Fukushi University.

*Correspondence to uchida@m.tohoku.ac.jp

The validation experiment of immunostaining for mouse anti-parvalbumin antibody with the mouse brain.

Free-floating sections were used for the validation experiment. The free-floating sections were rinsed in 10 mM phosphate-buffered saline (PBS; pH 7.4). For PV immunostaining, the sections were washed with PBS containing 0.1% (w/v) Triton X-100 (PBS-T), and then with incubated for 30 min with 1% normal donkey serum diluted in PBS-T. The sections were then incubated overnight at 4 °C with or without mouse anti-PV antibody (1:1000; clone No. PARV-19, catalogue No. P3088, Sigma-Aldrich Co.)). After washing with PBS-T, the sections were incubated with Alexa 555 donkey anti-mouse IgG (1:500; catalogue No. A-31570, Thermo Fisher Scientific, MA, USA) for 2 h at room temperature.

We were concerned about non-specific binding of the mouse-derived antibody; we performed the single staining of secondary antibodies. The sections incubated with mouse anti-PV antibody and Alexa 555 anti-mouse IgG indicated the accumulation of fluorescence signals in the specific neurons. The signals were strongly accumulated in the reticular thalamic nucleus, which abundantly contains PV. There were no signals in that the sections are incubated with 2nd antibody without anti-PV antibody (supple Fig. 1).

Heterogeneity of MeCP2 staining intensity was observed in the cortex of the hypothyroid group

While the transient downregulation of MeCP2 was observed in the cortex of the hypothyroid group, heterogeneous expression was caused in some areas. The phenomenon was observed in a region-nonselective manner, but the common site was found in the cortical layers II-IV. The heterogeneous expression was observed in 5 out of a total of 7 mice (supple Fig. 2). Ectopic expression is often observed in hypothyroid animals. Goodman et al. indicated thyroid hormone insufficiency leads a cellular malformation in the corpus callosum [1], and Lavado-Autric et al. reported the number of heterotopic BrdU-labelled cells are increased in the white matter [2]. These phenomena may be attributed to abnormalities in cell migration, but the heterogeneity in MeCP2 expression might be a transient change in the metabolic state of the cell. Further study was required on this point.

Expression of glutamate decarboxylase 1 (*GAD1*, GAD67) in the hippocampus and the cortex in the hypothyroid mice.

As described in the main text, mice (C57 BL/6J) were maintained under conditions of controlled temperature, relative humidity, and lighting. The control mice were fed laboratory chow with ad libitum tap water. To generate hypothyroid mice model, pregnant mice were administered antithyroid agents (see the main text) from post pregnancy day 17 to postpartum day 14. At postnatal day 14, mice under isoflurane anesthesia, mice were deeply anesthetized with three anesthetic agents (see the main text). The mice were transcardially perfused with 0.9% saline, and then by 4% paraformaldehyde (PFA) in 0.1 M phosphate buffer (PB; pH 7.4). Then, the brains were surgically obtained and fixed in 4% PFA at 4 °C for 6 h and immersed in 30% sucrose in 10 mM PB (pH 7.4) at 4 °C for 48 h until the brains sank to the bottom of the tube. Thirty micrometer-thick sections were serially cut on a cryostat, and the free-floating sections were rinsed in 10 mM phosphate-buffered saline (PBS; pH 7.4). For GAD67 immunostaining, the sections were washed with PBS containing 0.1% (w/v) Triton X-100 (PBS-T), and then with incubated for 30 min with 1% normal goat serum diluted in PBS-T. The sections were then incubated overnight at 4 °C with rabbit anti-GAD67 antibody (1:200, Nittobo Medical, Tokyo. JAPAN). After overnight incubation and washing with PBS-T, the sections were treated with 1% H_2O_2 to inactivate endogenous peroxidases. After another wash with PBS-T, the sections were incubated with biotinylated goat anti-rabbit IgG (1:1000; Vector Laboratories Inc., CA, USA) for 2 h at room temperature, rinsed with PBS-T, and incubated with avidin-biotin complex (ABC; Vector Laboratories Inc.) for 2 h at room temperature. Finally, the sections were visualized with 3,3'-diaminobenzidine (DAB).

For a Relative quantitative PCR (qPCR), total RNA was extracted from tissue homogenates and cDNA was synthesized. Each cDNA strand was amplified with Taq polymerase for qPCR, and the fluorescence intensity of each sample was detected using a LightCycler. Numerical analysis of semi-quantitative PCR was performed using Pfaffl's method (see the main text). Each primer sequence is as below.

GAD1 primer pair:
5'-GTGCAGGCTACCTCTTCCAG-3'
5'-CCACATCAGCCAGAACTTGA-3'

The Student's unpaired t-test was performed to compare the expression of *GAD1* between experimental groups. Data are shown as the means ± standard error of mean. Statistical differences were considered significant at $p < 0.05$.

GAD67 immunoreactivities were observed scattered in the cortex of mice at PD 14. In the hippocampus, GAD67 immunoreactive cells were observed in the hilus of the dentate gyrus, the stratum oriens, the stratum lacunosum-moleculare and the stratum radiatum. There were no differences in GAD67 immunoreactivities and expression of *GAD1* mRNA between the experimental groups (supple Fig. 3).

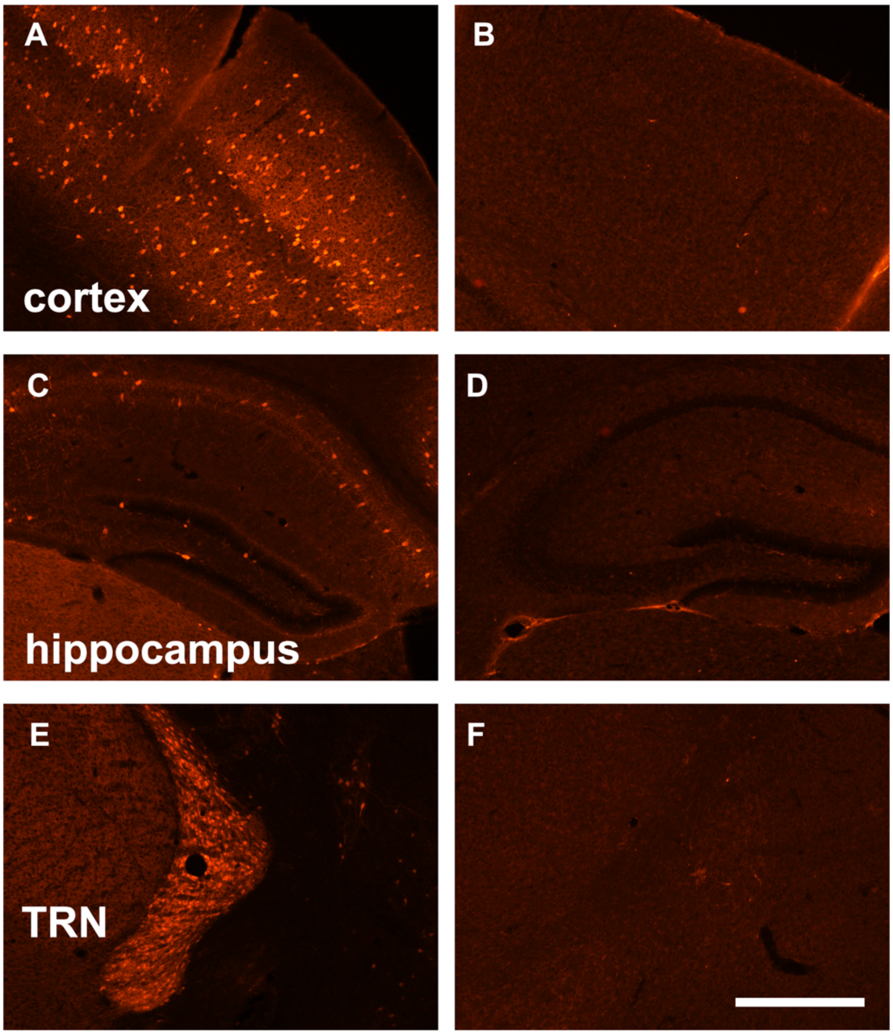

Supplemental Fig. 1

Immunostaining of mouse anti-parvalbumin antibody in the mouse brain. Photomicrographs show immunostaining of PV in the cortex (A), the hippocampus (C), and the thalamic reticular nucleus (TRN, E) of mous brain. Each right panel shows the result of single staining with 2nd antibody (B, D, and F). Scale bar = 500μm.

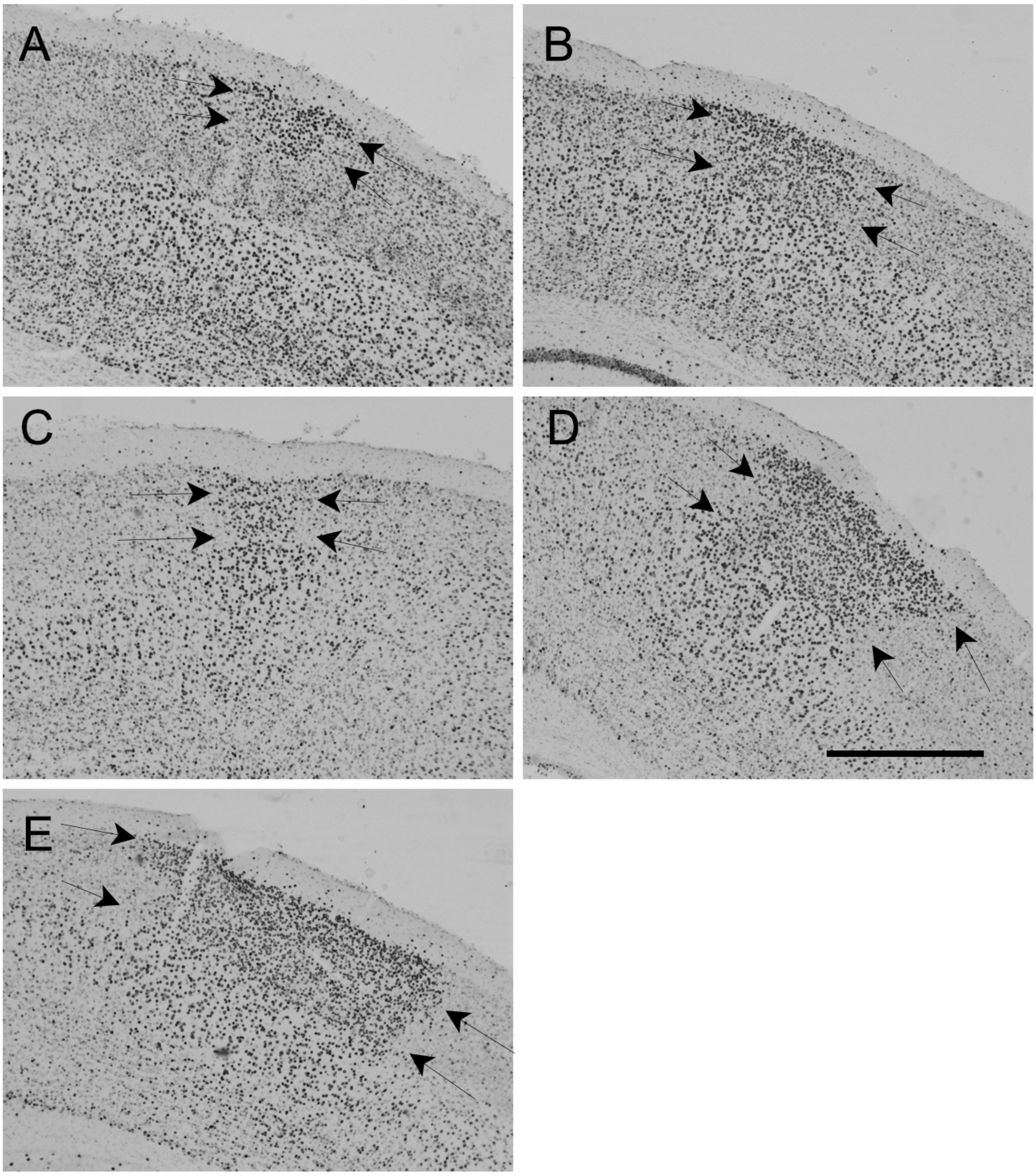

Supplemental Fig. 2

Heterogeneity of MeCP2 staining intensity in the cortex of the hypothyroid mice.

Each photomicrograph shows an image of the cortex of an individual mouse (A-E). Arrows indicate the areas of heterogeneity expression of MeCP2. Scale bar = 500μm.

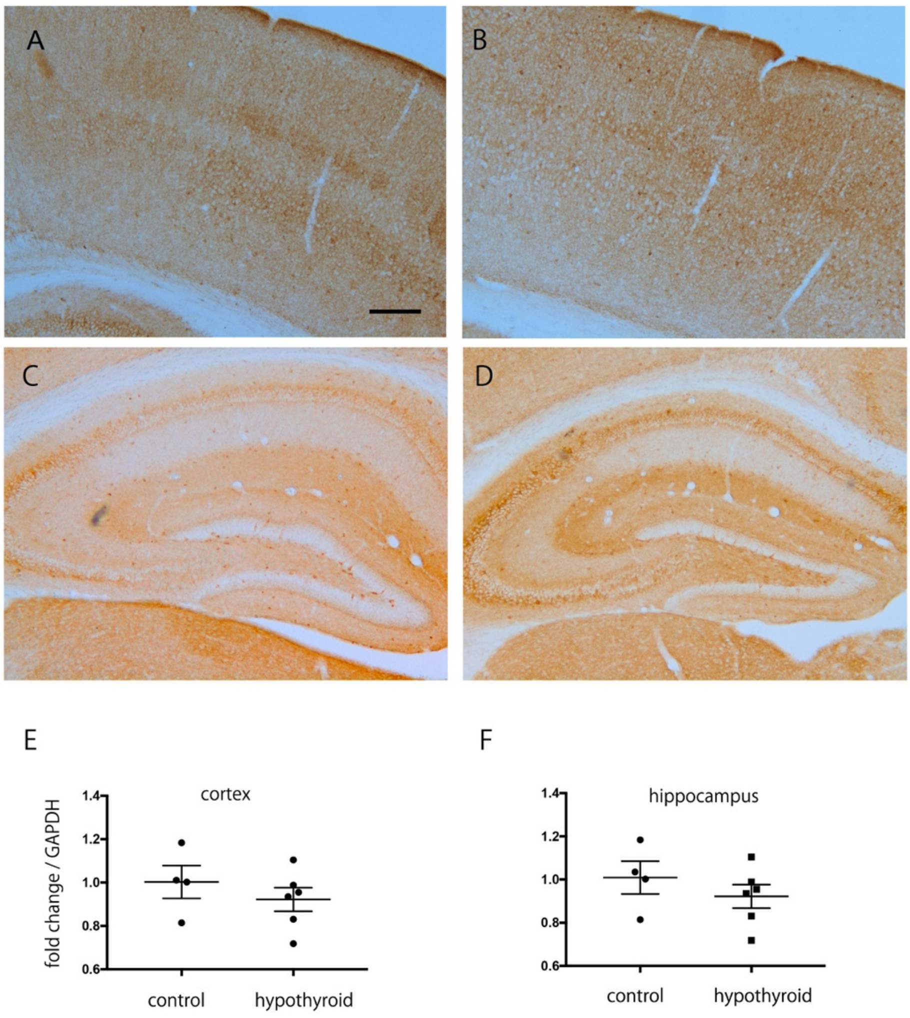

Supplemental Fig. 3

Expression of *GAD1* and GAD67 in the hippocampus and cortex of the mice.

Photomicrographs show immunostaining of GAD67 in the cortex (A, B) and the hippocampus (C, D) of normal and hypothyroid mice at PD 14, respectively. Scale bar = 200μm. Scatter plots show the expression levels of *GAD1* mRNA in the cortex (E) and the hippocampus (F) of the mice. Data are shown as the means ± SEM.

References

1 Goodman, J. H. & Gilbert, M. E. Modest thyroid hormone insufficiency during development induces a cellular malformation in the corpus callosum: a model of cortical dysplasia. *Endocrinology* **148**, 2593–2597, doi:10.1210/en.2006-1276 (2007).

2 Lavado-Autric, R. *et al.* Early maternal hypothyroxinemia alters histogenesis and cerebral cortex cytoarchitecture of the progeny. *J Clin Invest* **111**, 1073–1082, doi:10.1172/JCI16262 (2003).
